# News and Events of the Week

**Published:** 1910-10-08

**Authors:** 


### News and Events of the Week.

The Lord Mayor of Cardiff has received the following communication from Sir Arthur Bigge :—

Balmoral Castle.

To the Right Hon. the Lord Mayor of Cardiff.

The King has heard with interest and satisfaction of the generous contribution already made towards the campaign against tuberculosis in Wales, adopted as a National Memorial of the Principality to King Edward. Such an effort to combat the terrible disease always strongly appealed to his late Majesty, and cannot fail equally to enlist the hearty sympathy of the King.—A. Bigge.

The wardenship of Lyddon Hall, Leeds, which became vacant by the death of Mr. G. H. Rowe, has been filled by the appointment of Professor J. Kay Jamieson, M.B., C.M.

Recent German statistics show that the death-rate from diphtheria in Germany has gradually risen. In 1905 the average per 100,000 inhabitants was 24; in 1908 it had increased to 25.

The next meeting of the Society for the Study of Inebriety will be held in the rooms of the Medical Society of London, 11 Chandos Street, Cavendish Square, W., on Tuesday, October 11, at 4 p.m., when a discussion will be opened on "Alcohol and Primitive Races." Each member and associate is at liberty to introduce a visitor.

The remarkable mediæval poem "The Kingis Quair" (a favourite of D. G. Rossetti's), which is believed to have been written by King James I. of Scotland, is shortly to be published by Messrs. A. and C. Black. The volume has been edited by Professor Alexander Lawson, of St. Andrews University, and his introductions to "The Kingis Quair" and the "Quare of Jelusy," which was found in the same manuscript, discuss the authenticity and place of the former in mediæval Scottish and English literature, besides dealing in detail with the life of King James I. The poem is taken from the Bodleian Manuscript, Arch. Selden, B 24, as well as an amended text, and will be furnished with explanatory notes and a glossary.

As the result of a meeting of the United Kingdom Branch of the Association of Medical Women in India, to hear from Miss Benson, M.D., first physician of the Cama Hospital, Bombay, about some proposals to form an organised female medical service for India, and to receive information on the present working conditions of the Dufferin Fund, it was decided unanimously that (1) the Secretary of the Countess of Dufferin's Fund should be a qualified medical woman (not as at present a layman); (2) at least one qualified medical woman should have a seat on the Central Committee of same fund (and not only the President, Lady Minto); (3) an efficient service of medical women for India should be organised (at present there is no organisation at all). To further these objects it was resolved that the Secretary of State for India and Lady Hardinge (president-elect of the Dufferin Fund) should be asked to receive some members of the Association.

The Charing Cross Hospital Huxley Entrance Scholarship of fifty guineas has been awarded to Mr. R. V. Bevan, and the Universities Scholarship of fifty guineas to Mr. C. J. Marshall.

The public examination in bankruptcy of Mr. Robert Barclay Allardice, some time Mayor of Lostwithiel, and claimant to a Scottish earldom, was closed at Truro last Saturday. Mr. Allardice said he was a member of the American medical profession, but the American degree did not entitle him to practise in England. There were no estates that would follow the earldom if he obtained it, and it was due to Stock Exchange speculation that he was in his present position.

The minimum annual subscription to the Society for the Study of Inebriety being a merely nominal one (five shillings, including copy of the *British Journal of Inebriety*, post free), the Council have resolved that a reserve fund be established to further the work of the Society, and every member and associate and all interested in the scientific study of alcoholism are earnestly invited to forward contributions to the Hon. Treasurer, G. Basil Price, M.D., 42 Welbeck Street, Cavendish Square, London, W.

The following are the arrangements for the week at the Medical Graduates' College and Polyclinic, 22 Chenies Street, W.C. : Monday, October 19, at 4 p.m., Mr. J. E. R. McDonagh, Clinique (Skin); 5.15 p.m., Dr. Nestor Tirard, "Some Cases of Diabetes." Tuesday, at 4 p.m., Dr. Essex Wynter, Clinique (Med.); 5.15 p.m., Dr. George Pernet, "Syphilitic Headaches." Wednesday, at 4 p.m., Mr. A. Pearce Gould, Clinique (Surg.); 5.15 p.m., Mr. W. Stuart-Low, "The Nasal and Operative Treatment of Asthma." Thursday, at 4 p.m., Dr. Leonard Guthrie, Clinique (Med.); 5.15 p.m., Dr. F. J. McCann, "On some Common Mistakes in the Diagnosis of Cancer of the Uterus." Friday, at 4 p.m., Dr. Dundas Grant, Clinique (Ear, Nose, and Throat).

At the Congregation held on the opening of the new academical year at Cambridge on October 1, the outgoing Vice-Chancellor delivered a valedictory address to the Senate, in the course of which he announced that the Drapers' Company had offered to build a new physiological laboratory similar in cost (£22,000 and £1,000 for fittings) to the fine electrical laboratory which they have lately bestowed on the University of Oxford. "I need not say," proceeded Canon Mason, "that the University is in no way pledged beforehand to accept the offer. The Worshipful Company are aware that the acceptance will mean the raising on our part of a sum which may be roughly calculated at £5,000 or £6,000 for upkeep, and that we have no superfluous money at hand for these things. They know that if we hesitate before we accept, it is not because we fail to admire the princely magnitude of their offer, or are ungrateful for their renewed kindness towards us, but only because it is necessary for us carefully to consider ways and means."

The freedom of the city of Dublin has been conferred upon Sir Charles Alexander Cameron, the public analyst of the city and head of the Public Health Department. To his efforts members of the corporation attributed the comparative freedom of Dublin from almost all malignant diseases.

Their Majesties the King and Queen have graciously consented to become Patrons of the Royal National Mission to Deep-Sea Fishermen, as did Queen Victoria and Queen Alexandra before them. The Queen has also consented to the naming of the Mission's next hospital steamer the *Queen Mary*.

The following is a list of the Child Study Society's lectures and discussions for 1910 to be held at 90 Buckingham Palace Road, S.W. Thursday, October 13, at 7.30 p.m., "Some First Results of an Investigation into the Play Interests of English Elementary School Children," by Miss Alice Ravenhill: Chairman, Sir James Crichton-Browne, M.D., LL.D., F.R.S. Thursday, October 27, at 7.30 p.m., "Games and Toys for Children under Eight," by Miss Clara E. Grant: Chairman, C. W. Kimmins, D.Sc., M.A. Thursday, November 3, at 7.30 p.m., "Story of some Children's Games," by Mrs. Lawrence Gomme: Chairman, Professor J. Adams, M.A. Thursday, November 17, at 7.30 p.m., "The Origin of Certain Games and Toys" (illustrated by lantern slides), by A. C. Haddon, M.A., D.Sc., F.R.S.: Chairman, H. Holman, M.A. Thursday, November 24, at 7.30 p.m., "Philosophy of Boys' Games," by Felix Clay, F.R.I.B.A.: Chairman, James Kerr, M.A., M.D. Thursday, December 1, at 7.30 p.m., "The Child's Inheritance," by C. W. Saleeby, M.D., F.R.S. Edin.; Chairman, Sir Edward Brabrook, C.B.

Royal Society of Medicine, 15 Cavendish Square, W., Thursday, October 6, 8 p.m., Obstetrical and Gynæcological Section, at 11 Chandos Street, W. *Demonstrations* (*with epidiascope*): Dr. Victor Bonney and Mr. Bryden Glendining: "Adenomatosis Vaginæ." Dr. Macnaughton-Jones: "Sarcoma of a Polypus of the Cervix Uteri" (shown for Professor Schottlaender). *Short communication*: Dr. Drummond Robinson: "Some Notes on the Vaginal Secretion of Infants." Presidential address by Sir Wm. Church: "The lessons of a session."

At Marylebone County Court last Tuesday Dr. W. Peart-Thomas, of Leinster Square, sued the Duke of Manchester for £85 for professional services rendered. It appeared that the Duke had instructed the plaintiff to take charge of a demonstration on four consumptive patients at a nursing home. The Duke alleged that the treatment of the patients was experimental, and that the plaintiff was only asked to attend in order to give a death certificate if necessary. Judge Selfe held that the plaintiff had been placed in charge of the experiments, and gave judgment in his favour for £63, with costs.

Acrimonious public correspondence between two medical men in Sydney (writes a Melbourne correspondent) has made prominent the differences that exist between the Australian Medical Association and the N.S.W. branch of the British Medical Association. The members of the latter Society refuse to meet members of the former, who assert that they are boycotted. The British Associates' objection to the Australians is that they either refuse to join the Medical Union or hold appointments in friendly Societies below the rates allowed, thus acting in a manner detrimental to the interests of the profession.

The second International Conference for the Study of Cancer was opened last week by M. Doumergue, Minister of Public Instruction, in the Great Hall of the Medical Faculty at the Sorbonne, under the presidency of Professor Czerny.

The annual festival of the Guild of St. Luke (Birmingham Ward) will be held on Tuesday, October 18, at 4.15 p.m., in Worcester Cathedral, when the sermon will be preached by the Very Rev. the Dean of Worcester. All medical practitioners are invited.

Saturday last, October 1, was the anniversary of the death of the Earl of Shaftesbury, the first President of the Ragged School Union and Shaftesbury Society. Following the custom of previous years, a wreath was placed on the statue of the great philanthropist in Westminster Abbey.

The Lettsonian lectures of the Medical Society of London will be delivered on February 6 and 20 and March 6, 1911, by Mr. Wm. Haslam, F.R.C.S., on "A Review of the Operations for Stone in the Male Bladder." The anniversary dinner will take place at the Whitehall Rooms, Hôtel Métropole, on Wednesday, March 8, 1911, at 7.30 p.m.

The farewell meeting of the Zenana Bible and Medical Mission (founded 1852) will be held in the Throne Room, Holborn Restaurant, on Tuesday, October 11, 1910, at 3 p.m. Chairman, The Lord Kinnaird. The farewell address to the missionaries will be given by the Rev. F. S. Webster, M.A.

Several women are taking the four years' course of study, chiefly chemistry, for the profession of food analyst in Australia, but the first to emerge from studentship is Miss Myrie Phillips Shappere, an Australian-born girl of Polish parentage. Food analysis would seem to be peculiarly suitable to women, since the qualities required include patience, perseverance, cleanliness, carefulness, and a capacity for details.

The following are the arrangements for next week at the West London Postgraduate College: October 10 and 13, at 10 a.m., Demonstration by Surgical Registrar; October 14, at 10 a.m., Demonstration by Medical Registrar; October 11, at 11.30 a.m., Demonstration of Minor Operations; October 10, at 12 noon, Pathological Demonstration; October 12, at 12.15 p.m., Dr. Grainger Stewart, Practical Medicine. Lectures at 5 p.m.: October 10, opening address, by Donald W. C. Hood, C.V.O., M.D., F.R.C.P., subject "Specialism in Medicine"; October 11, Dr. Saunders, Clinical Lecture with Cases; October 12, Dr. Beddard, Practical Medicine; October 13, Mr. Baldwin, Practical Surgery; and October 14, Dr. Saunders, Clinical Lecture with Cases.

